# Research on the Application of Artificial Intelligence in Public Health Management: Leveraging Artificial Intelligence to Improve COVID-19 CT Image Diagnosis

**DOI:** 10.3390/ijerph20021158

**Published:** 2023-01-09

**Authors:** Tiancheng He, Hong Liu, Zhihao Zhang, Chao Li, Youmei Zhou

**Affiliations:** 1 Department of Political Party and State Governance, East China University of Political Science and Law, Shanghai 201620, China; 2 Teacher Work Department of the Party Committee, Shanghai University of Traditional Chinese Medicine, Shanghai 201203, China; 3 College of Computer Science and Technology, Shanghai University of Electric Power, Shanghai 200090, China; 4 Department of Computer Science, Zhijiang College of Zhejiang University of Technology, Hangzhou 310024, China; 5 Department of Landscape Architecture, College of Architecture and Urban Planning, Tongji University, Shanghai 200092, China

**Keywords:** public health, COVID-19, artificial intelligence, automatic segmentation, CT image diagnosis

## Abstract

Since the start of 2020, the outbreak of the Coronavirus disease (COVID-19) has been a global public health emergency, and it has caused unprecedented economic and social disaster. In order to improve the diagnosis efficiency of COVID-19 patients, a number of researchers have conducted extensive studies on applying artificial intelligence techniques to the analysis of COVID-19-related medical images. The automatic segmentation of lesions from computed tomography (CT) images using deep learning provides an important basis for the quantification and diagnosis of COVID-19 cases. For a deep learning-based CT diagnostic method, a few of accurate pixel-level labels are essential for the training process of a model. However, the translucent ground-glass area of the lesion usually leads to mislabeling while performing the manual labeling operation, which weakens the accuracy of the model. In this work, we propose a method for correcting rough labels; that is, to hierarchize these rough labels into precise ones by performing an analysis on the pixel distribution of the infected and normal areas in the lung. The proposed method corrects the incorrectly labeled pixels and enables the deep learning model to learn the infected degree of each infected pixel, with which an aiding system (named DLShelper) for COVID-19 CT image diagnosis using the hierarchical labels is also proposed. The DLShelper targets lesion segmentation from CT images, as well as the severity grading. The DLShelper assists medical staff in efficient diagnosis by providing rich auxiliary diagnostic information (including the severity grade, the proportions of the lesion and the visualization of the lesion area). A comprehensive experiment based on a public COVID-19 CT image dataset is also conducted, and the experimental results show that the DLShelper significantly improves the accuracy of segmentation for the lesion areas and also achieves a promising accuracy for the severity grading task.

## 1. Introduction

Since the start of 2020, the outbreak of the Coronavirus disease (COVID-19) has been a globally public health emergency, and has caused unprecedented economic and social disaster [1]. It features a number of symptoms including endothelial barrier disruption, dysfunctional alveolar-capillary oxygen transmission, reduced oxygen diffusion capacity, alveolar wall thickening, increased vascular permeability and pulmonary oedema [2]. As a major global public health emergency, COVID-19 has once again proved that human beings live in a “global risk society” with a common destiny, reminding us to be more alert to new and recurrent infectious diseases and to build a strong public health system to provide strong protection for people’s health. There is no doubt that the development of COVID-19 has exceeded most people’s expectations. Because of the rapid spread of COVID-19, the timely detection of the COVID-19 infection is essential to carry out the prompt isolation and treatment of COVID-19 patients. At present, reverse transcription-polymerase chain reaction (RT-PCR) is the most widely adopted method for the detection of COVID-19. However, RT-PCR suffers from several limitations: (1) it is time-consuming (requiring over 3 h to complete the detection process); (2) there is limited supply of test kits; (3) poor sampling quality causes false negatives [3]. Nowadays, CT plays an important role for detecting COVID-19 [3], and bilateral patchy shadows or ground-glass opacity in the lung can be clearly identified from chest-computed tomography (CT) images captured from COVID-19 patients [4]. In addition, compared with RT-PCR, the operation of chest CT is easy, and with chest CT we can judge the severity of the disease. Therefore, CT could serve as a practical method for the diagnosis of COVID-19. Moreover, to assess the severity of COVID-19, contouring the infected area is an essential procedure for an image diagnosis. However, the traditional manual contouring operation is tedious and time-consuming, and it heavily depends on the clinical experience of physicians. With the increase in the number of infected patients, the workload of radiologists has significantly increased; hence, an automatic CT image segmentation method for COVID-19 diagnosis is urgently expected.

Deep learning technology has been widely adopted in medical image segmentation due to its capability of feature extraction [5]. Deep learning methods show excellent performance in the task of the lesion segmentation of COVID-19, but large-scale labelled samples must be available prior to applying these deep learning methods [6,7,8,9]. The task of collecting sufficient COVID-19 CT images and accurately labelling them at a pixel level is time-consuming and costly. To tackle this issue, some methods employ data augmentation [10,11] and image synthesis [12,13] to extract the information from limited labeled images, but they usually suffer from poor generalization on different datasets. Other methods applying semi-supervised [14,15] and unsupervised learning [16,17] fail to achieve good performance due to the large variations of infection on CT images, such as irregular shapes and ambiguous boundaries [18].

Not only the quantity but also the quality of the pixel-level label restricts the training process of deep learning methods. By reviewing these dominant public COVID-19 CT image datasets, we found that: (1) the quality of the dataset is uneven because it is susceptible to the experience of the physician; and (2) the translucent ground-glass characteristics of the infected area are hard to accurately identify, and further lead to the labeling of some non-infected areas as infected areas (such as lung parenchyma and pulmonary vessels). In this study, we aim to correct these mislabeled labels to provide well-labelled datasets for model training. Rough labels (with mislabeled ones) are hierarchized according to the pixel distribution of the infected and normal areas in the lung image. The proposed method reassigns the mislabeled pixels and enables the deep learning model to learn the infected degree of each infected pixel. With the hierarchical labels, we propose a deep learning-based aiding system (named DLShelper) for COVID-19 diagnosis. The DLShelper performs lesion (in lung) segmentation from CT images, as well as the task of severity grading. A multi-layer preceptor (MLP) is used as a classifier. The proportion of the lesion to the lung and the proportion of each grade in the lesion are used as input features. Rich auxiliary diagnostic information (e.g., the severity grade, the proportion of the infected area and the visualization of the infected area) are provided for the physicians in clinic. The main contributions in this paper are as follows:

- In order to improve the performance of segmentation on COVID-19 infection, a label refinement method is proposed to refine the existing labels from rough to precise. The refinement reassigns the incorrectly labeled pixels and enables the network to learn the infection degree of each infected pixel.
- Aiming to assist physicians in the efficient diagnosis of COVID-19, a deep learning-aided system (named DLSHELPER) using refined hierarchical labels is proposed. DLSHELPER provides rich auxiliary diagnostic information, including the proposed severity grade, proportion of infected area and infected area visualization.
- We validate the accuracy of our method for COVID-19 lesion segmentation and grading on public COVID-19 CT datasets.

At present, it is an important opportunity to change the public health governance system. This study takes the diagnosis of COVID-19 as an example to explore the enabling effect of AI in the management of public health emergencies.

The rest of the paper is organized as follows: Section 2 introduces the related work. Section 3 details the proposed method for COVID-19 CT image diagnosis. Section 4 presents the experiment and discussion. Finally, Section 5 concludes the study.

## 2. Related Work

In recent years, the intelligent analysis of medical images based on artificial intelligence has been extensively researched [19]. Santosh et al. [20] proposed a lung feature detection model based on multi-feature parameters, and it achieved an accuracy of up to 91%. Pratondo et al. [21] combined multiple machine learning models and a region-based contouring algorithm for the task of medical image segmentation. Ahmad et al. [22] used the Content-Based medical image retrieval algorithm for lung segmentation. However, its Jaccard similarity coefficient was only 0.870. Shepherd et al. [23] proposed a statistical model based on shape prior for segmentation combined with online/offline learning models. Xu et al. proposed a method for lung function assessment based on cough sound [24]. Shaukat et al. [25] developed a fully automated method to detect lung nodules using a hybrid feature set of SVM and achieved a promising accuracy. Souza et al. [26] proposed a Deep Convolutional Neural Network method (DCNN) for fully automated lung segmentation. Park et al. [27] used DCNN for lung CT image segmentation. Although DCNN is capable of learning complex data, it is overly dependent on the amount of data used in the training process. Besides, the size of the data also impacts the performance of the model.

The quality of these public CT image datasets is uneven because they are susceptible to the experience of the physician. In addition, in contrast to the semantically segmented objects, the COVID-19 lesion is translucent and of a low contrast with the surroundings. The labeling operation is conducted manually; hence, the labeling process unavoidably involves human errors. Some normal pixels are mislabeled as infected ones in the situations where: (1) the lung parenchyma pixels are entrapped between lesion pixels; (2) other areas are tissues such as pulmonary vessels. These mislabeled pixels will weaken the performance of model training.

## 3. Method

### 3.1. Overview

**Figure 1:**
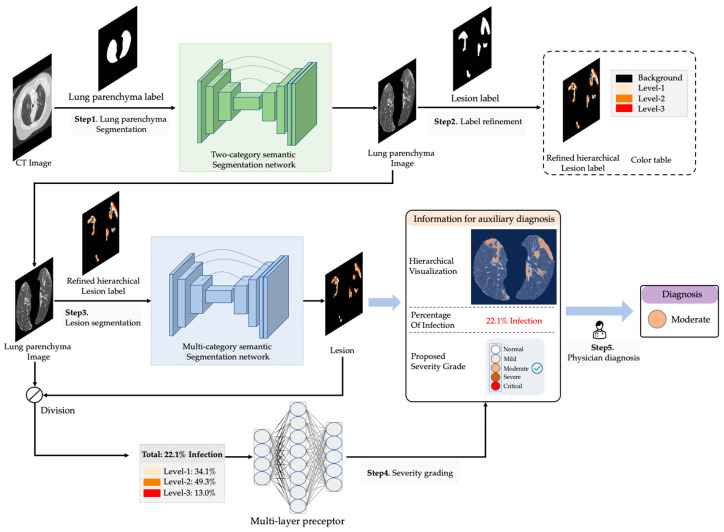
The full workflow of the proposed auxiliary diagnosis system.

As shown in Figure 1, the functions of the proposed method include: (1) lung segmentation; (2) lesion label refinement; (3) lesion segmentation; and (4) severity grading. The original CT images and lung parenchyma labels are used to train a two-category semantic segmentation network, which is used for segmenting the lung parenchyma image. With these segmented lung parenchyma images and lesion labels, the infected and normal areas can be identified. By further analyzing the pixel distribution in these two areas, the mislabeled pixels can be corrected and these pixels can be hierarchized to different levels according to the value of each pixel so that these rough lesion labels are finally refined to accurate hierarchical labels. The “level” not only represents the value of a pixel, but also indicates the infected degree of the area in which the pixel is contained. The lung parenchyma images and refined hierarchical labels are used to train a multi-category semantic segmentation network, then we use it to segment the lesion areas. Different output lesions are converted into different colors to generate a hierarchical visual map that provides intuitive information for auxiliary diagnosis. We calculate the proportion of three categories in the lesion area, respectively. Then, the total proportion of the lesion to the whole lung parenchyma is provided as other information for auxiliary diagnosis. Moreover, these four radiological features are used as input parameters for the severity grading, which is based on a three-layer multi-layer preceptor (MLP). In summary, there are three types of information provided to physicians by the proposed system: (1) the hierarchical visual map; (2) the proportion of the lesion in the lung area; and (3) the severity grade.

### 3.2. Label Refinement

As discussed in Section 2, with the traditional method pixels marked as infected may contain normal pixels. Moreover, the traditional strategy for lesion labeling only includes the categories: infected (marked as 1) and normal (marked as 0), which ignores the information contained in the infected pixels; e.g., for each pixel in the infected area, the higher the value, the more serious the infection is.

**Figure 2:**
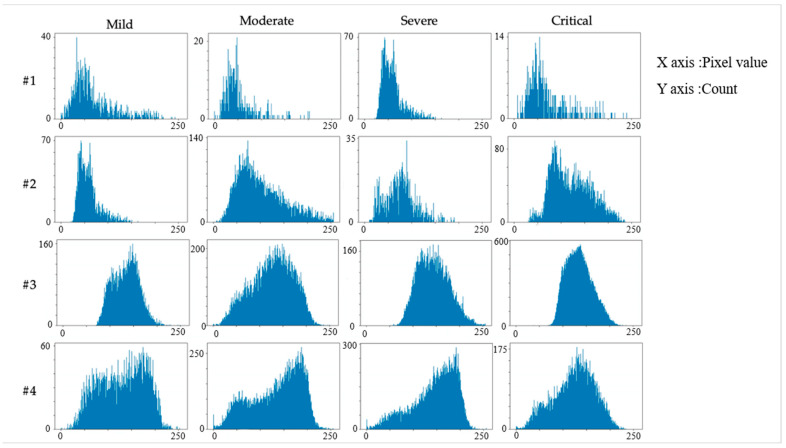
The comparison of the grayscale histograms of four CT images (lesions) from different severity grades.

For two lesions with the same area (assuming that the area of the lung parenchyma in which they are located is also equal and the value of pixel falls in the range (0–255)), the more the grayscale distribution approaches 255, the more serious the infection is in clinical diagnosis. As shown in Figure 2, we selected four CT images from different severity grades and calculated grayscale histograms of their lesions. The results reveal a positive correlation between the grayscale distribution of the lesion with its severity. However, there is no accurate metric to measure the grayscale distribution, Therefore, we hierarchize the infected area to a different level according to its pixel value, and the grayscale distribution can be described by the percentage of pixels at different levels in the lesion.

We denote the CT image as I and its corresponding lesion label as M. M has the same size as I. We obtain the lung pixel from I by applying lung segmentation and denote it as $O_{Lung}$. Then, the lesion in I is obtained by the mask operation in I and M, and we denote it as $O_{Infected}$. The complement of $O_{Infected}$ in $O_{Lung}$ is $O_{No\text{-}infected}$ (the normal pixel in the lung). These processes are formulized as:

(1) $$I_{Lung}=N_{Two\text{-}catagory}\left(I\right)\;⊗I,\;O_{Lung}=\{p\in \;I_{Lung}\;|\;I_{Lung}\left(p\right)>0\}\;$$

(2) $$I_{Infected}=I\;⊗M,\;O_{Infected}=\{p\in \;I_{Infected}\;|\;I_{Infected}\left(p\right)>0\}$$

(3) $$O_{No\text{-}infected}={∁}_{O_{Lung}}O_{Infected}$$

where $N_{Two\text{-}catagory}$ denotes the network for lung parenchyma segmentation, ⊗ denotes the element-wise multiplication and p denotes the pixel in the image; ${∁}_{a}b$ denotes the complement of a in set B.

We denote the average value of $O_{No\text{-}infected}$ as a and the maximum of $O_{Infected}$ as b, respectively. Given the pixel of $O_{Infected}$ can be divided into Grade-g, the pixel with a value less than a and greater than or equal to Grade-g will be reassigned as the background. These processes are formulized as:

(4) $$a=\mathrm{Mean}\left(O_{No\text{-}infected}\right)$$

(5) $$b=\mathrm{Max}\left(O_{Infected}\right)$$

**Figure 3:**
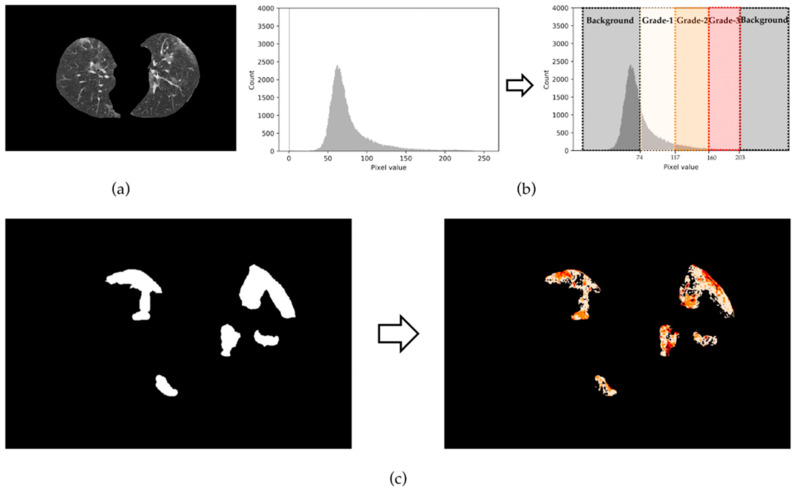
Visualization of label refinement (g = 3): (**a**) is the lung image segmented from a CT image. The left part of (**b**) is the histogram of (**a**) and we grade the pixels based on the pixel distribution of the infected and uninfected areas in the lung; (**c**) shows the lesion labels before and after the refinement.

(6) s=(b−a)/(g+1)

(7) $$R_{0}\in \left[0,\;a\right]\cup \left(b-s,\;255\right]$$

(8) $$R_{i}\in \;(a+\left(i-1\right)*s,\;a+i*s],0<i\leq g$$

where s denotes the interval between grades. $R_{0}$ represents the range of pixel values of the background; meanwhile, $R_{i}$ represents the range of pixel values of Grade-i (0<i≤g). Finally, we assign the pixel belonging to each grade in $I_{Lung}$; i.e., the value of the Grade-i pixel is set to i;the value of the background pixel is set to 0. Thus, a refined hierarchical label is generated. Of note, the reason for the pixel with a value greater than or equal to Grade-g being reassigned as the background is that lung trachea and blood vessels may be contained in these pixels. A value of g that is too small or too large will impact the accuracy of the label refinement; hence, we use it as a hyper-parameter and compulsorily set it to 3 (according to experimental results). As shown in Figure 3c, mislabeled infected pixels are corrected to normal ones, and these infected pixels are hierarchized to different grades.

### 3.3. Lung and Lesion Segmentation

Traditional segmentation models (especially UNet [28]) have achieved good performance on segmentation tasks for lung and COVID-19 lesions. UNet adopts symmetric encoding and decoding paths to aggregate semantic information and recover spatial information with the help of shortcut connections, and it is suitable for medical image segmentation. Thus, in this study, we adopt UNet for lung and lesion segmentation. In addition, we use a multiple-category training strategy (instead of the traditional two-category strategy) to learn the grades of pixels in the lesion.

With the completion of network training, a CT image will be input to the UNet to segment a lung image. Then, the obtained lung image is input to the multiple-category segmentation network to obtain the COVID-19 lesion. Based on these different categories of lesions, a colorful visualized map is generated hierarchically.

### 3.4. Severity Grading

As reported in [26], the number, quadrant and area of lesions in CT images are important factors to determine the severity of the COVID-19 case. However, as the area of lung parenchyma in a volume of continuous CT image slices is different, it is inappropriate to use a fixed value as the threshold to determine the grade of severity. In this work, we calculated the proportion of all lesions in the lung parenchyma to address this issue. We found that the higher the value, the whiter the pixel appears in the lesion area, so the level of the “white” pixel in the lesion area and the density of the white pixel can be taken as indicators for determining the grade of severity. With regard to these indicators, a multilayer perceptron (MLP) is used as a classifier for severity grading.

**Figure 4:**
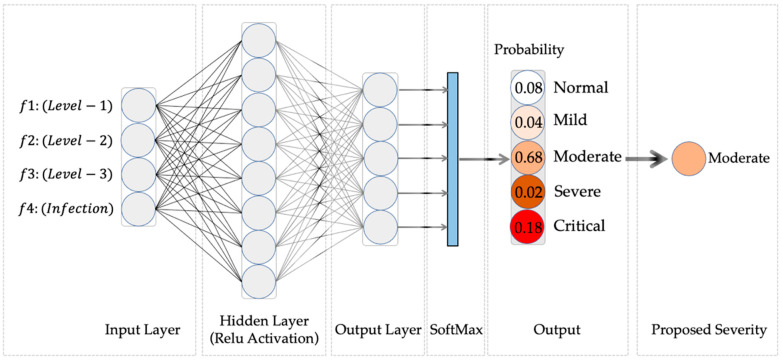
Structure of the multi-layer preceptor network (g = 3). $f_{1–4}$ denotes the input features of the MLP network. $f_{1–3}$ denotes the proportion of infected pixels of three levels in all infected pixels. $f_{4}$ denotes the proportion of infected pixels in lung parenchyma pixels.

The multilayer perceptron is a feedforward artificial neural network that uses supervised back-propagation, which is widely used for nonlinear classifications. As shown in Figure 4, the MLP in the proposed method consists of an input layer, a hidden layer and an output layer. The Relu function is used as the activation function of the hidden layer and the SoftMax function is used as the activation function of the output layer for the classification. The number of neurons in the hidden layer is determined by an empirical formula:

(9) $$k=\sqrt{m+n}+a$$

where k denotes the number of neurons in the hidden layer, n denotes the number of neurons in the input layer, m denotes the number of neurons in the output layer, and a denotes a constant between 1 and 10.

## 4. Experiments and Analysis

The dataset [29] used in this study contains about 3500 CT image slices and corresponding lung and lesion segmentation labels. In addition, we recruited a radiology graduate student to label each CT image with a grade of severity (e.g., normal, mild, moderate, severe and critical). The labels were then verified by an experienced radiology specialist for reliability.

### 4.1. Implementation and Evaluation

A two-stage training strategy is adopted in this experiment: (1) training the segmentation of lung and COVID-19 lesions; (2) oversampling the training set, and finally training the MLP. We reproduced all the related networks and modules in the Pytorch framework. When training the segmentation network, we set the number of the batch size to 1, then initialize the network weights with Kaiming initialization, set network biases to zero and train the positive/negative samples alternately. In addition, the training set is shuffled in each iteration. We use different metrics in different stages. Intersection over union (IoU), sensitivity (SEN), specificity (SPE) and Dice similarity coefficient (DSC) are used to evaluate the accuracy of the lung segmentation. Besides, mean intersection over union (mIoU), mean pixel accuracy (mPA) and class pixel accuracy (CPA) are used to evaluate the accuracy of COVID-19 lesion segmentation using original labels and refined hierarchical labels. Precision is used to evaluate the accuracy of the severity grading. The above-mentioned metrics can be calculated as:

(10) $$\mathrm{IoU}\;=\frac{\mathrm{TP}}{\mathrm{TP}+\mathrm{FP}+\mathrm{FN}}$$

(11) $$\mathrm{SEN}\;=\frac{\mathrm{TP}}{\mathrm{TP}+\mathrm{FN}}$$

(12) $$\mathrm{SPE}\;=\frac{\mathrm{TN}}{\mathrm{TN}+\mathrm{FP}}$$

(13) $$\mathrm{DSC}\;=\frac{2\mathrm{TP}}{2\mathrm{TP}+\mathrm{FP}+\mathrm{FN}}$$

(14) $$\mathrm{mIoU}=\frac{1}{k+1}\sum_{i=0}^{k}\frac{\mathrm{TP}}{\mathrm{FN}+\mathrm{FP}+\mathrm{TP}}$$

(15) $$\;\mathrm{Precision}=\frac{\mathrm{TP}}{\mathrm{TP}+\mathrm{FP}}$$

(16) $$\mathrm{mPA}\;=\frac{1}{k+1}\overset{k}{\underset{i=0}{\sum }}\frac{\mathrm{TP}}{\mathrm{TP}+\mathrm{FP}}\;$$

where TP denotes true positives, TN denotes true negatives, FP denotes false positives and FN denotes false negatives.

We optimize the lung and lesion segmentation networks using a binary cross-entropy $L_{lung}$ and a multi-category cross-entropy loss $L_{lesion}$, respectively, using a mean-squared error loss to train the MLP $L_{mlp}$.

(17) $$L_{lung}\left(a,b\right)=-\left[blog\left(a\right)+\left(1-b\right)\log\left(1-a\right)\right]$$

(18) $$L_{lesion}\left(a,b\right)=-\overset{g}{\underset{m=0}{\sum }}b_{m}logf{\left(a\right)}_{m}\;$$

(19) $$f{\left(a\right)}_{m}=\frac{e^{a_{m}}}{\sum_{n=0,\;n\neq m}^{g}e^{a_{n}}}\;$$

(20) $$L_{mlp}\left(a,b\right)={\left\|a-b\right\|}^{2}$$

(21) $$L=L_{lung}+L_{lesion}+L_{mlp}$$

where *a* is the ground truth and *b* is the predicted result. *g* is the number of grades in refined hierarchical labels.

### 4.2. Evaluation of Lung Segmentation

**Figure 5:**
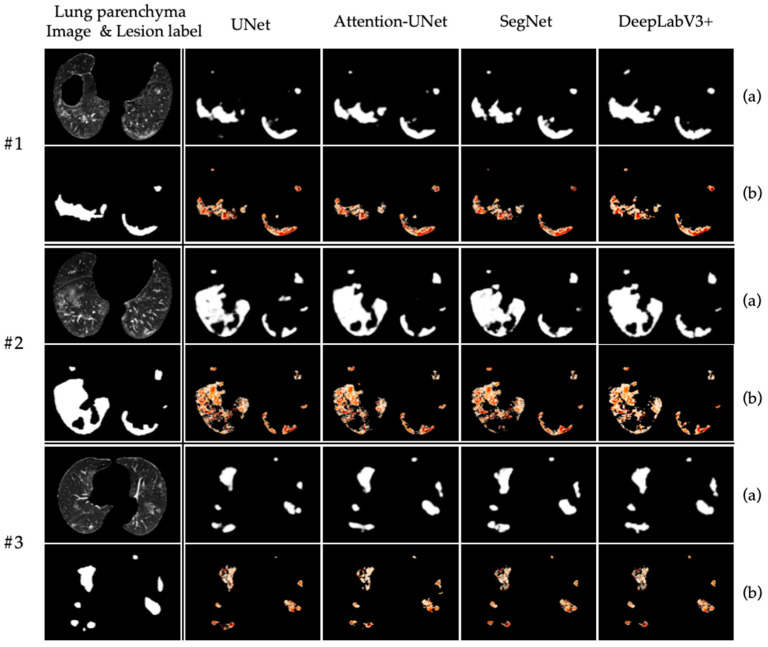
Visualization of the lesion segmentation using original label and refined hierarchical label. (**a**) and (**b**) illustrate the segmentation results using original lesion labels and refined hierarchical labels, respectively.

As shown in Table 1 and Figure 5, lung segmentation with UNet works efficiently, with DSC up to 96%. Besides, IoU, SEN and SPE all surpass 90%. The accurate segmentation of the lung parenchyma ensures the quality of subsequent COVID-19 lesion segmentation and severity grading.

### 4.3. Evaluation of COVID-19 Lesion Segmentation Using Refined Hierarchical Labels

To evaluate the performance of refined hierarchical labels for COVID-19 lesion segmentation, four state-of-the-art networks are selected and trained with original labels and refined hierarchical labels, respectively (as shown in Figure 5). With regard to these widely used metrics (e.g., IoU, DSC, SEN and SPE) for medical image segmentation, an evaluation is carried out. Table 2 shows the values of these four metrics of the model trained with original labels and refined hierarchical labels. Table 3 shows the values of these four metrics, the CPA of each level and the MIoU and MPA of the model trained with refined hierarchical labels.

With the original labels, DeepLabV3+ achieves the best DICE of 82.94% among all the networks. Meanwhile, UNet achieves the worst performance. However, we find that the area marked as a lesion by the original labels in the input image contains many normal pixels such as lung parenchyma and pulmonary vessels. As illustrated in Figure 5, the #2 image is the most mislabeled. By introducing the refined hierarchical labels, the segmentation network can not only accurately identify the infected pixels, but also filter out these mislabeled pixels. Besides, as shown in Table 2, with the introduction of refined hierarchical labels, the model achieves better performance, such as the DSC of UNet and Attention-UNet reaching 83.47% and 82.35%, respectively. As shown in Table 3, the performance of pixel segmentation with UNet (2) is the best. Because the ground truth used is different, we cannot directly compare the performance of models training on original labels and refined hierarchical labels. Experienced radiologists from a hospital in Zhejiang Province confirm that refined hierarchical labels bring more precise results.

### 4.4. Evaluation of COVID-19 Severity Grading

There are few samples of mild, severe and critical cases in the dataset. As shown in Table 4, the classification accuracy of these categories is very low, even as low as 0 (mild). To solve this category imbalance problem, we applied the Synthetic Minority Oversampling Technique (SMOTE) [33] to minority classes. With the operation of oversampling the dataset, all the categories of samples reached a balance, the classification accuracy of mild reached 100% and the classification accuracy of severe and critical increased by 19.81% and 10.25%, respectively. Moreover, the overall accuracy was 98.82%.

## 5. Conclusions

In this study, we propose a method for refining lesion labels from rough to precise. Then, a deep learning-based aiding system for CT image diagnosis using refined labels is developed. It performs lung and lesion segmentation from CT images, as well as severity grading. A multi-layer preceptor is used as a classifier, and the proportion of the lesion to the lung and the proportion of each grade in the lesion are used as input features. Auxiliary diagnostic information including the severity grade, proportion of infected area and visualization of the infected area are provided by the DLShelper for physicians in clinic. A comparative experiment based on public datasets is carried out, and the experimental results show that the proposed method achieves better accuracy in comparison with several state-of-the-art networks. Besides, the proposed method achieves a high accuracy for severity grading. In future, we will develop a new metric to describe the grayscale distribution features so as to further improve the performance.

In COVID-19 prevention and control, while developing AI and playing its positive role, we should be alert to the social risks and ethical challenges brought by AI itself, carry out responsive and principled scientific and technological governance, and strengthen ethical review and data legislation under the principle of “harmony, friendship, fairness, inclusiveness and sharing, respect for privacy, security and controllability, shared responsibility, open cooperation, and agile governance”.

## Figures and Tables

**Table 1:** Obtained result of lung segmentation metrics.

Metric	IoU	DSC	SEN	SPE
Value	0.94	0.96	0.97	1.00

**Table 2:** Comparison of lesion segmentation performance using original labels and refined hierarchical labels.

Network	Labels		IoU (%)	DSC (%)	SEN (%)	SPE (%)
	Original	Refined				
UNet [28]	√		68.02	78.36	82.31	99.74
		√	73.54	83.47	87.75	99.83
Attention-UNet [30]	√		71.89	82.13	85.51	99.78
		√	71.82	82.35	82.37	99.87
SegNet [31]	√		68.98	79.57	83.38	99.74
		√	68.49	79.54	78.85	99.87
DeepLabV3+ [32]	√		72.43	82.94	85.08	99.79
		√	68.71	80.48	79.89	99.84

**Table 3:** Detailed performance using refined hierarchical labels.

Network	g	CPA (%)	IoU (%)	DSC (%)	SEN (%)	SPE (%)	MIoU (%)	MPA (%)
UNet	1	77.30	56.52	70.71	77.30	99.87	61.04	75.73
	2	81.31	65.47	77.20	81.31	99.92		
	3	75.33	61.13	72.93	75.33	99.95		
Attention-UNet	1	70.30	53.24	67.80	70.30	99.88	58.27	71.56
	2	73.95	62.82	75.17	73.95	99.94		
	3	70.41	58.75	70.81	70.41	99.96		
SegNet	1	55.58	41.31	56.20	55.58	99.84	51.96	67.74
	2	71.21	52.55	66.77	72.22	99.86		
	3	66.59	49.40	63.18	66.59	99.92		
DeepLabV3+	1	55.21	40.62	56.20	55.21	99.85	41.69	57.05
	2	61.62	44.43	60.37	61.62	99.86		
	3	54.34	40.03	55.11	54.34	99.91		

**Table 4:** Precision of Severity Grading.

Severity	Precision (%)	Precision (%) (With Operation of Oversampling)
Normal	1	1
Mild	0	1
Moderate	99.37%	99.38%
Severe	62.69%	82.50%
Critical	79.49%	89.74%
Total	96.49	98.82

## Data Availability

Data are available by request to the corresponding authors.
